# MRI for patients with cardiac implantable electronic devices: simplifying complexity with a ‘one-stop’ service model

**DOI:** 10.1136/bmjqs-2018-009079

**Published:** 2019-02-13

**Authors:** Anish N Bhuva, Patricia Feuchter, Angela Hawkins, Lizette Cash, Redha Boubertakh, Jane Evanson, Richard Schilling, Martin Lowe, James C Moon, Charlotte H Manisty

**Affiliations:** 1 Department of Cardiac Imaging, Barts Heart Centre, Barts Health NHS Trust, London, UK; 2 Institute of Cardiovascular Sciences, University College London, London, UK; 3 Department of Radiology, Barts Health NHS Trust, London, UK; 4 Department of Cardiac Electrophysiology, Barts Heart Centre, Barts Health NHS Trust, London, UK

**Keywords:** MRI, CIED, health inequality, teamwork, Quality improvement

## Abstract

**Background:**

Patients with cardiac pacemakers and defibrillators are disadvantaged because of poor access to MRI scans, leading to late and misdiagnosis particularly for cancer and neurological disease. New technology allied to tested protocols now allows safe MRI scanning of such patients; however, logistical barriers persist.

**Aim:**

To deliver a streamlined sustainable service that provides timely MRI scans to patients with cardiac implantable electronic devices (CIEDs).

**Methods:**

Patients requested a ‘one-stop’ service for MRI, whereby devices could be reprogrammed and scans acquired at a single location and visit. To provide this ‘one-stop’ service, we trained a team including administrators, physicians, cardiac physiologists and radiographers. A standard protocol was used to prevent unnecessary request refusals and delays to scheduling. Service volume, waiting time and safety were analysed 6 months before and 2 years after service redesign. Waiting times for internal and external inpatient referrals plus time to treatment for patients on a cancer pathway were analysed.

**Results:**

215 MRI scans were performed over 2 years. After service redesign, MRI provision increased six-fold to 20 times the national average with reduced waiting time from 60 to 15 days and no adverse events. Departmental throughput was maintained. 85 (40%) referrals were external. 41 (19%) inpatients were scanned, reducing bed-stay by 3 days for internal referrals. 24 (11%) scans were for suspected cancer, 83% allowed treatment within the national standard of 62 days. There was no preintervention service for either inpatients or suspected cancer investigation.

**Conclusion:**

Implementation of a ‘one-stop’ service model to provide MRI for patients with CIEDs is safe, streamlined, scalable and has reduced delays making economic and clinical sense. Protocols and checklists are available at mrimypacemaker.com.

## Introduction

Nearly half a million people in the UK have cardiac pacemaker or defibrillators,^1^ each with a 75% lifetime chance of needing a MRI scan.^2^ Clinicians are increasingly dependent on MRI for diagnosis and management of many acute, severe conditions (cancer, stroke, radiotherapy planning, spinal cord compression), and it is the fastest growing imaging modality in the UK.^3^ Patients with cardiac pacemakers or defibrillators (collectively termed cardiac implantable electronic devices, CIEDs) are likely to benefit the most because they are older (one in 50 people over 65 have a pacemaker) and have more comorbities.^4^ Unfortunately, they have been prevented from having MRI scans due to historical safety concerns, resulting in more invasive, inaccurate testing and delayed treatment. There is now a large evidence base showing these scans can be performed safely following appropriate protocols.^5^ Manufacturers have also made software and hardware modifications to develop ‘MRI-conditional’ CIEDs designed to undergo MRI. This makes scanning technically straightforward, but despite annual UK investment of over £100 million in MRI-conditional CIEDs, patients still cannot access MRI scans because services are logistically difficult to provide.^6–8^

At our centre, we witnessed first-hand the clinical need and consequences of underprovision. We therefore addressed logistical barriers and aimed to deliver a streamlined sustainable service that provides timely MRI scans to patients with CIEDs.

## Methods

### Logistical considerations

MRI scanning for patients with CIEDs requires some additional steps to be taken both prior to imaging and on attendance. After a referral is made, it is necessary to confirm whether the CIED is MRI-conditional. This can be done via patient records, device identification cards held by the patient or referrers. Logistics are similar if there is a non MRI-conditional CIED, but a clear indication is established in advance through a risk-benefit discussion.

Device checks must also take place before and immediately after the MRI scan to check device integrity and to programme into MRI-mode. This is done either by a cardiac physiologist or cardiologist and requires a portable device programming unit. There is also a safety checklist to ensure that there are no exclusions to scanning (eg, recent device implantation).

During the scan, the patient is monitored using at least one of ECG or pulse-oximetry, and an external defibrillator with pacing capability should be available within the department. Scanning is performed using lower specific absorption rate power following manufacturer recommendations. On MRI completion, the device is restored to the initial settings after interrogation—either in the cardiology department or near the scanner.

### Logistical barriers

Prior to redesigning our service, we surveyed all hospitals in England performing MRI to understand obstacles to service establishment, with an 86% response rate.^7^ Although 98% were aware of MRI-conditional devices, less than half provided scans to device patients, with only three performing more than 20 scans annually. Reported barriers to provision were primarily logistical and educational. There is limited cardiology and radiology interaction at physician and technician level, and so centralising a service that requires different parts of the hospital to work together is difficult. Because of a lack of training, clinicians also reported high perceived risk and safety concerns.

### Local context

Two adjacent 1.5T MRI scanners within the cardiac imaging department were used for this project (in addition to a 3T scanner not used for patients with CIEDs). In total, the department performs more than 7500 scans annually. This is within a large teaching hospital providing specialist tertiary referral cardiology and oncology services, which is paired with three large general hospitals. A full range of both cardiology and radiology services are available on-site. Prior to intervention, scans for patients with CIEDs were scanned with ad-hoc liaison between cardiology and radiology services.

Locally, we surveyed patient experience and referral time. Of 15 patients with CIEDs surveyed after receiving an MRI appointment, 7 (47%) reported being denied an MRI scan at their local hospital because they had a pacemaker (even though MRI-conditional). 11 (73%) reported delays in receiving appointments; including two with waits of over 2.5 years.

### Setting and intervention

**Figure 1:**
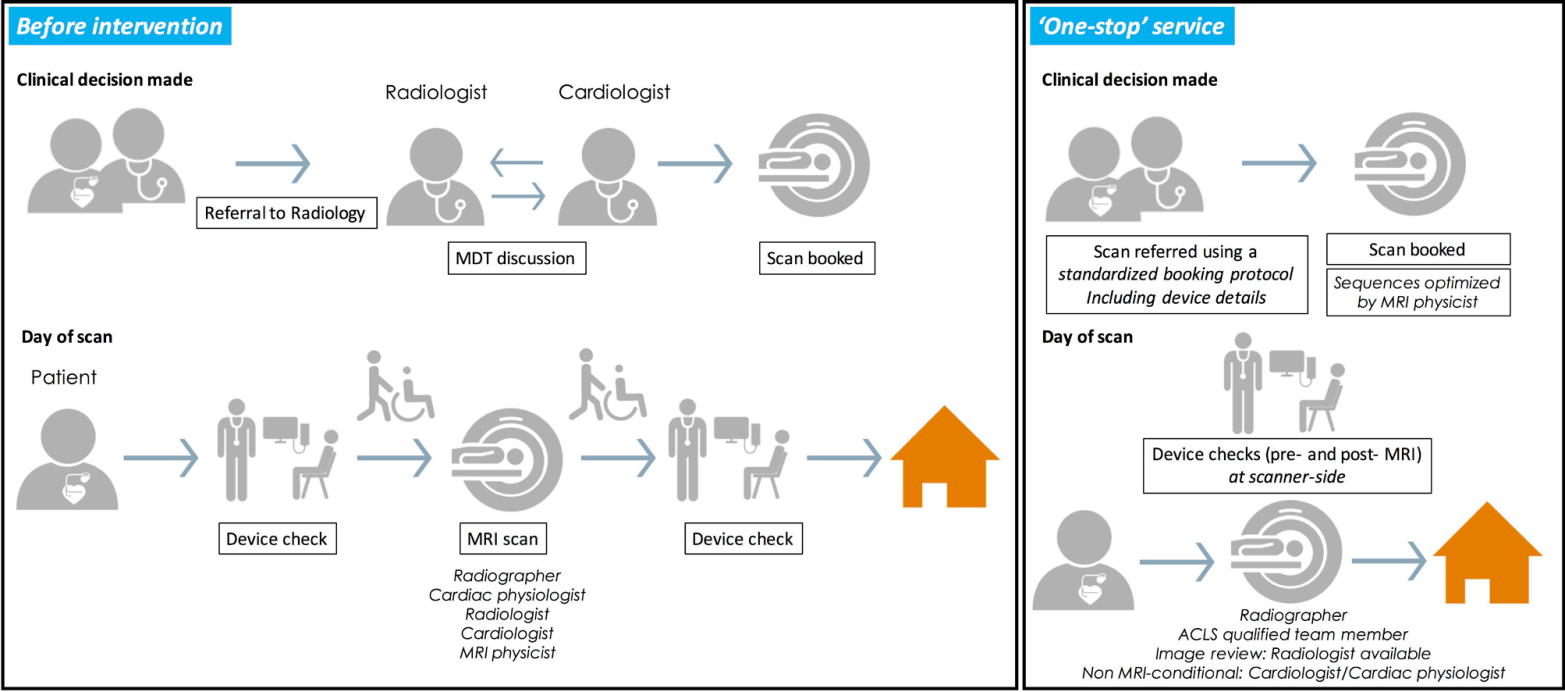
Service redesign into a ‘one-stop’ service. ACLS, advanced cardiac life support.

Patients and referrers requested a ‘one-stop’ service for MRI, whereby devices could be reprogrammed and scans acquired at a single location on a single visit. To provide a streamlined service (figure 1), we trained a team of named individuals including administrators, physicians, cardiac physiologists and radiographers and developed a standard booking protocol. This was designed to prevent unnecessary request refusals, prevent delays both to scheduling and during scanning and so improve efficiency, patient experience and safety. Because the team usually works in different areas of the hospital, we organised bookings into preallocated scanning sessions for patients with CIEDs. These changes allowed all necessary staff to be present for the scan, and meant specific individuals could be trained to perform the service. No additional equipment or other fixed costs were needed.

With time, the model also gained economies of scale: radiographers felt confident to scan patients with MRI-conditional CIEDs without cardiologist supervision in the control room, and pathways were developed for streamlining external referrals and more complex non MRI-conditional devices. As the project developed, we adjusted pathways to improve patient experience—feedback from a paraplegic patient, for example, drove changes in the logistics of difficult interhospital transfers.

### Measures of improvement

The primary outcomes were the volume of service provision; waiting time (receipt of referral to scan date) and safety as defined in the Magnasafe registry.^9^ These were measured at quarterly intervals 6 months before and 2 years after service redesign in January 2016. Inpatients and patients with a suspected cancer diagnosis were felt to be important groups.^6 9^ As there was minimal service provision before intervention, there were no baseline metrics of quality improvement for comparison. We therefore assessed whether scan results expedited inpatient flow by allowing discharge or determining procedural intervention. We also measured waiting time (clinical decision to scan date) for internal compared with external referrals. For patients with a suspected cancer diagnosis, we measured whether patients received treatment within the national standard of 62 days.^10^ Data were abstracted from hospital electronic medical informatics database including patient demographics, referral source, indication and follow-up management.

### Ethical considerations

According to the policy activities that constitute research locally, this work met criteria for operational improvement activities exempt from ethics review. The reporting of this quality improvement project was approved by the Institutional Quality Improvement Review Board and follows the proposed Standards for Quality Improvement Reporting Excellence guidelines.^11^

## Results

### Service volume and waiting time

**Figure 2:**
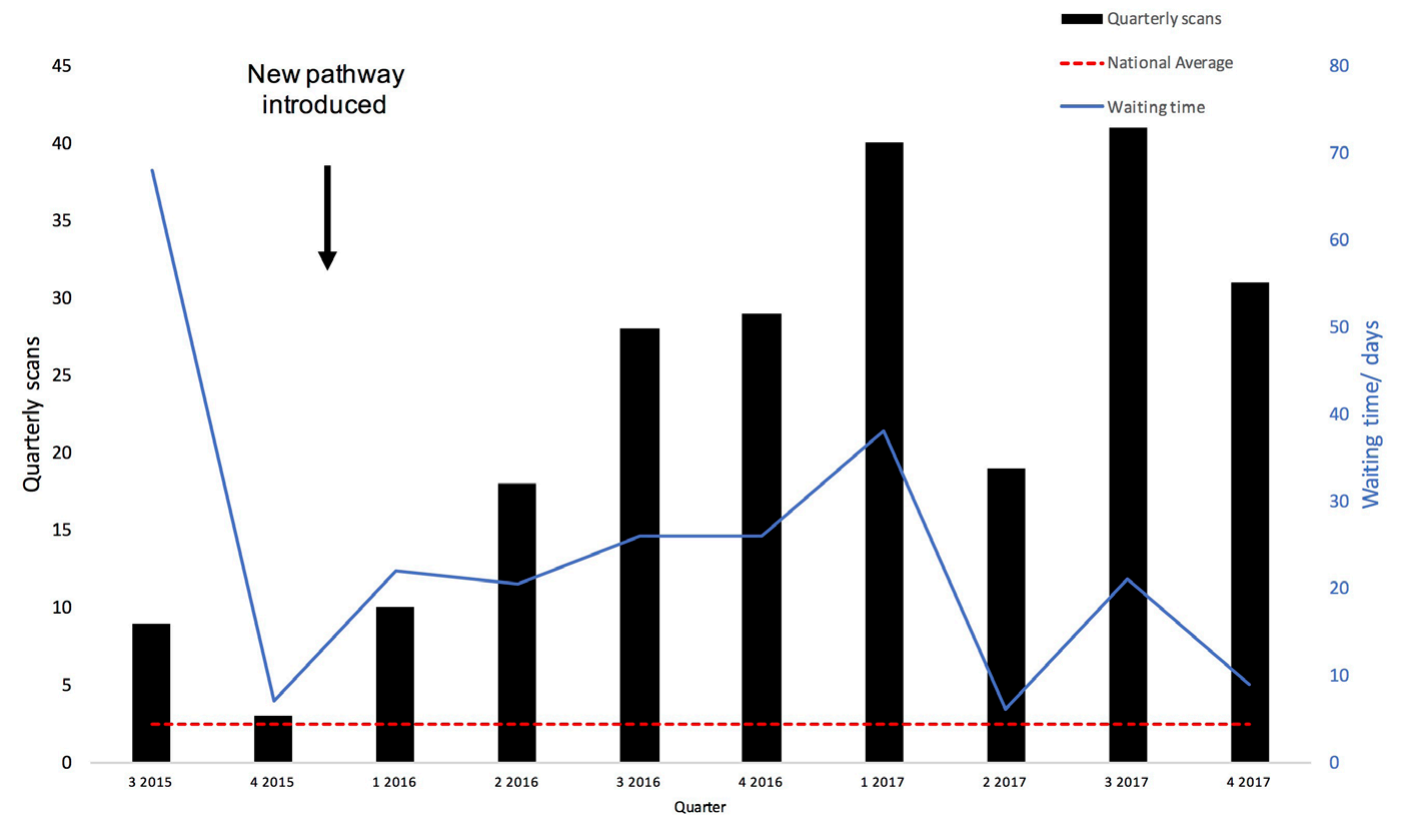
Quarterly rates of MRI provision and waiting time for patients with cardiac implantable electronic devices before and after new pathway introduction. Waiting time defined as time from receipt of referral to scan date. National average quarterly scan provision from Sabzevari *et al.* 7

Total 215 MRI scans were performed over 24 months for patients with CIEDs (age 61±20 years, 67% male, 19% hospitalised inpatients). In the 6 months prior to intervention, 6 scans/quarter were performed, increasing sixfold to 36 scans/quarter for the final 6 months. This is 20 times the national average, figure 2. Waiting time also reduced from 60 (IQR: 34–71) to 15 (IQR: 4–45) days. All MRI scans were performed safely with no adverse events, despite scanning patients with increasingly complex cardiac devices. All scans were performed for standard clinical indications often with no alternative imaging modalities. Given essentially absent service prior to the new model, all benefits are not merely incremental as patients would have had no access to standard NHS care. There was no reduction in total department activity.

### Inpatients and cancer investigation

Forty-one (19%) inpatients were scanned, and 32% were for urgent diagnoses (cancer, stroke, cord compression or life-threatening heart rhythms). Eighty-three per cent expedited patient flow (discharge or determining procedural intervention) and scans were three bed-days faster for internal than external referrals, table 1. Twenty-four (11%) scans were for a suspected cancer diagnosis, with an 8 (IQR: 0–32) day waiting time. Eighty-three per cent allowed treatment within the national standard of 62 days. There was no preintervention service for either inpatients or suspected cancer investigation.

**Table 1:** Waiting time in days for internal and external referrers

	*n*	Internal	External	Total
Overall	*215*	28 (7–60)	69 (16–107)	41 (8–79)
Inpatient	*41*	4 (1–10)	7 (1–17)	4 (1–13)
Outpatient	*174*	42 (16–116)	75 (39–66)	49 (22–80)

Data are represented as median (IQR). Waiting time defined as time from clinical decision to scan date.

### Referral location

**Figure 3:**
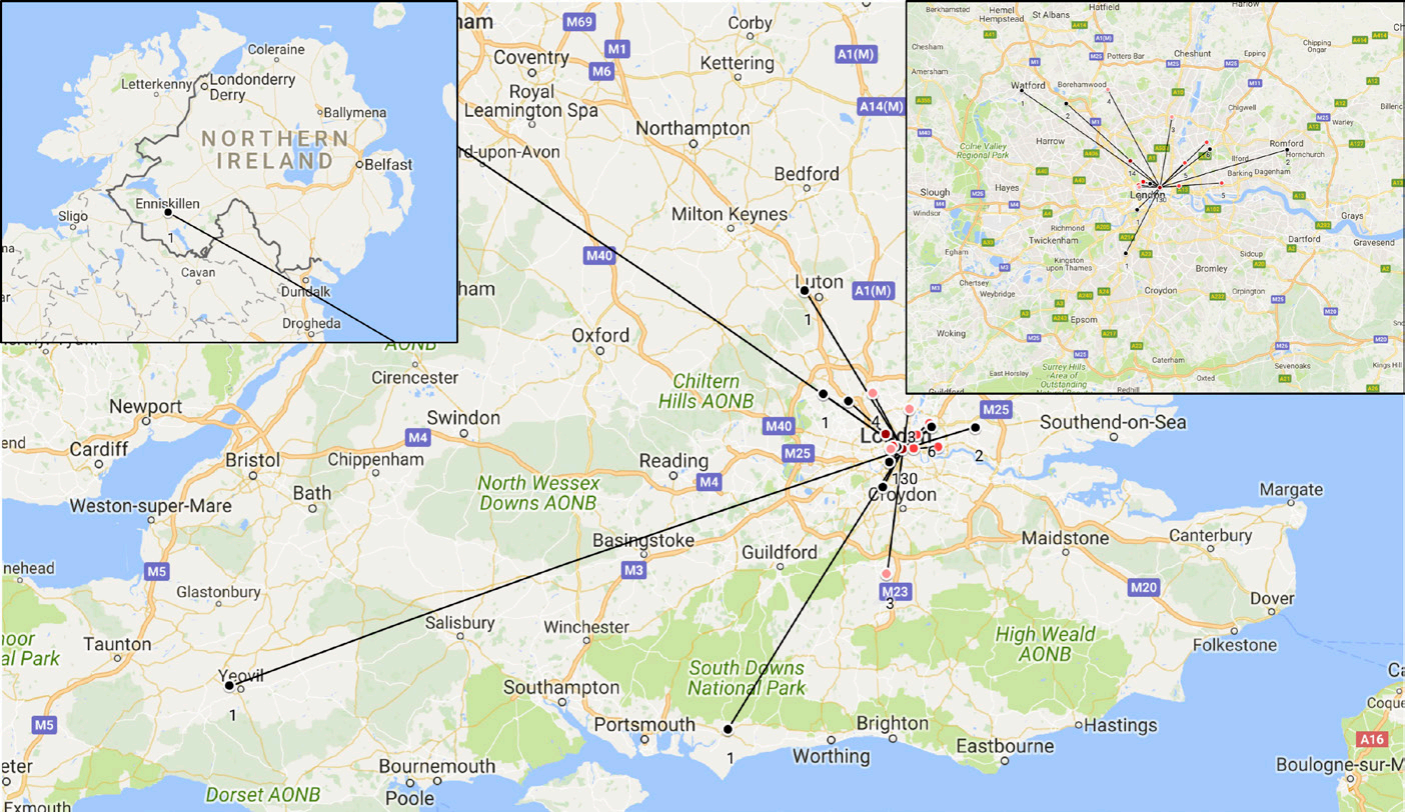
Catchment of external referrals to the newly established service.

Eighty-five (40%) referrals were from other hospitals and the service developed into a supraregional hub at an early stage (figure 3). Time from clinical decision to scan was 69 (IQR: 16–107) days for external referrals compared with 28 (IQR: 7–60) days for internal referrals.

## Discussion

We redesigned MRI provision for patients with CIEDs into a ‘one-stop’ service where devices could be reprogrammed and scans acquired at a single location on a single visit. International guidelines, MRI-conditional pacemakers and two large studies last year alone have highlighted this important but difficult issue.^5 9 12^ Based on our baseline data collection, we have shown that national provision is extremely low because of multiple barriers.^7^ There is lack of awareness among radiologists, cardiologists and referrers; logistical and funding barriers.^7 8^ In this context, we redesigned a service starting with a provision rate just above the national average and then became the largest UK centre and a supraregional hub.

By making scans easier to access, referrals increased six-fold but a streamlined model meant that the department was able to perform scans safely, more efficiently and at scale. After 2 years, scan volume was over 20 times the national average with a lower waiting time, and while maintaining total departmental activity. Before service redesign, patients with CIEDs could not access MRI, with delays in diagnosis of up to two and a half years. All benefits to patients were therefore not just incremental: making MRI available meant that patients were able to access standard NHS pathways. This was often for important indications such as diagnosis of stroke or spinal cord compression. One in 10 scans were for cancer and these patients frequently reattended (up to seven occasions) to guide surgical resection, enable radiotherapy or confirm remission.

### Breaking down silos of practice

This was achieved by creating change with evidence-based pathway redesign, introducing cultural change and new ways of working across silos of hospital practice. The need for new working practices to provide this service are recognised by the Royal College of Radiologists, British Cardiovascular Society and British Heart Rhythm Society guidelines.^13 14^ We specifically sought to understand and address barriers to service provision. Staff did not feel confident performing scans due to lack of training and so we concentrated experience in specific individuals. Logistically, it was difficult to collate the information needed to protocol scans. Training of the bookings team using a specific proforma similar to international guidelines ensured all decisions were made beforehand and delay was minimised at the scanner-side.^5^ By creating a transparent process, the team was able to approve scans faster and more frequently, but it required a learning curve to implement the process efficiently as represented by waiting time data figure 2. This should allay concerns of other services in coping with the increased demand. After concentrating experience in a few individuals, we could adapt our service model so radiographers performed some scans without supervision and the team were able to scan patients with increasingly complex devices with a pressing clinical indication safely (eg, CyberKnife planning). Radiographers were also able to ask for scan review for completeness by an appropriate imaging expert to avoid recall.

### Organisational benefits outweigh costs

Performing MRI scans for patients with CIED is in the best interests of the patient and the healthcare system. The diagnostic yield and clinical benefit from these scans is high—common requests are for suspected cord compression, stroke and cancer or planning for radiotherapy or neurosurgery. This is in agreement to other centres in the USA and Italy.^6 8^ Early diagnosis makes clinical and economic sense;^15^ the financial cost alone of a late cancer diagnosis is £4000 even when accounting for treatment costs, and an extra bed day is £222.^16 17^ This saving was most obvious for inpatients where bed stay reduced by 3 days for internal referrals. In the short term, prompt decision making meant early patient discharge (£17 300 saving on internal referrals alone) and alternative suboptimal investigations avoided. This underestimates the benefit because this was compared with external referrals who also would not have had access to MRI prior to service redesign. In the medium to long term, health savings accrue with downstream cost savings of reduced population morbidity and associated social care.

The changes involved were primarily organisational and logistical, incorporating better interdisciplinary communication. Modest funding for device checks and staff time on the scan day was needed. MRI-conditional devices are now standard of care in the UK, meaning the additional cost is already invested at device implantation. Eighty-five per cent of departments nationally currently have the necessary equipment available.^7^

### A growing clinical need

We were surprised at the frequency of external referrals from across the UK for patients unable to access scans locally. The organic growth of our service into a supraregional hub even without advertising highlights the clinical urgency to develop this nationally. While unique, the knowledge and skills to provide this model elsewhere are scalable and may offer a template which does not significantly impact on resources. We also hope such a model can be used for other multidisciplinary services necessary to deliver increasingly complex patient care.

A broader network approach, however, is needed to address this nationally. Ninety-four per cent of all MRI departments have cardiology on site and therefore could offer similar provision.^7^ Once a clinical decision had been made, it took externally referred patients 41 days longer to receive a scan. This was because patients generally were booked for and refused scans locally, before our unit was approached. Information related to patients’ devices was also often not available to us, leading to delays in collating the necessary information. To encourage other centres, direct contacts for scanning centres, national training courses, safety checklists and standard operating procedures are freely available online at *mrimypacemaker.com*.

### Limitations

Comparisons were made between internal and external referrals because of limited service prior to intervention and so improvements are likely to be underestimated. We have found that other local champions have faced their own barriers to service development which will depend on the individual microenvironment. It requires time to prepare for each scan which requires adequate administration resources and funding. The benefits observed are likely to be a combination both of service reorganisation with the ‘one-stop’ pathway, but also better awareness among referring clinicians and service providers. The relative contributions of these two factors are not known. Our service requirements continue to rise and this presents new challenges. Creating a network of scanning centres and establishing national tariffs that acknowledge the complexity will help to address this.

## Conclusion

A service model for MRI provision to pacemaker and cardiac defibrillator patients is sustainable at scale. This is achieved by creating a protocol to reduce scheduling delays and a ‘one-stop’ visit for patients with the multidisciplinary team. Rapid service growth highlights the pressing clinical and economic benefits of making MRI more available to cardiac device patients. Template standard operating procedures and checklists are available at mrimypacemaker.com

## References

[1] National Institute of Cardiovascular Outcome Research National audit of cardiac rhythm management devices, 2016.

[2] Kalin R, Stanton MS Current clinical issues for MRI scanning of pacemaker and defibrillator patients. Pacing Clin Electrophysiol 2005;28:326–8. 10.1111/j.1540-8159.2005.50024.x 15826268

[3] NHS England Operational Information for Commissioning Diagnostic imaging dataset. Available: https://did.hscic.gov.uk [Accessed 23 Jun 2017].

[4] Bradshaw PJ, Stobie P, Knuiman MW, et al Trends in the incidence and prevalence of cardiac pacemaker insertions in an ageing population. Open Heart 2014;1:e000177 10.1136/openhrt-2014-000177 25512875 PMC4265147

[5] Indik JH, Gimbel JR, Abe H, et al 2017 Hrs expert consensus statement on magnetic resonance imaging and radiation exposure in patients with cardiovascular implantable electronic devices. Heart Rhythm 2017;14:e97–153. 10.1016/j.hrthm.2017.04.025 28502708

[6] Nazarian S, Roguin A, Zviman MM, et al Clinical utility and safety of a protocol for noncardiac and cardiac magnetic resonance imaging of patients with permanent pacemakers and implantable-cardioverter defibrillators at 1.5 Tesla. Circulation 2006;114:1277–84. 10.1161/CIRCULATIONAHA.105.607655 16966586 PMC3410556

[7] Sabzevari K, Oldman J, Herrey AS, et al Provision of magnetic resonance imaging for patients with 'MR-conditional' cardiac implantable electronic devices: an unmet clinical need. Europace 2017;19:425–31. 10.1093/europace/euw063 27256417

[8] Celentano E, Caccavo V, Santamaria M, et al Access to magnetic resonance imaging of patients with magnetic resonance-conditional pacemaker and implantable cardioverter-defibrillator systems: results from the really ProMRI study. Europace 2018;20:1001–9. 10.1093/europace/eux118 29016759

[9] Russo RJ, Costa HS, Silva PD, et al Assessing the risks associated with MRI in patients with a pacemaker or defibrillator. N Engl J Med 2017;376:755–64. 10.1056/NEJMoa1603265 28225684

[10] Department of Health Next steps on the NHS five year forward view, 2017. 10.1136/bmj.j1678 28377430

[11] Ogrinc G, Davies L, Goodman D, et al Squire 2.0 (standards for quality improvement reporting excellence): revised publication guidelines from a detailed consensus process. BMJ Qual Saf 2016;25:986–92. 10.1136/bmjqs-2015-004411 PMC5256233 26369893

[12] Nazarian S, Hansford R, Rahsepar AA, et al Safety of magnetic resonance imaging in patients with cardiac devices. N Engl J Med 2017;377:2555–64. 10.1056/NEJMoa1604267 29281579 PMC5894885

[13] British Heart Rhythm Society Standards for implantation and follow-up of cardiac rhythm management devices in adults, 2018. 10.15420/aer.2024.01 PMC11287656 39082056

[14] Kmietowicz Z Patients with cardiac devices should not be excluded from MRI scans, say experts. BMJ 2018;362 10.1136/bmj.k3623 30135066

[15] Royal College of General Practitioners, Society and College of Radiographers, The Royal College of Radiologists Quality imaging services for primary care: a good practice guide, 2013.

[16] National Institute for Health and Care Excellence (NICE) Transition between inpatient hospital settings and community or care home settings, 2015.

[17] Cancer Research UK Saving lives, averting costs, 2014.

